# A Bioactive Fraction from *Streptomyces* sp. Enhances Maize Tolerance against Drought Stress

**DOI:** 10.4014/jmb.2003.03034

**Published:** 2020-05-13

**Authors:** Mona Warrad, Yasser M. Hassan, Mahmoud S.M. Mohamed, Nashwa Hagagy, Omar A. Al-Maghrabi, Samy Selim, Ahmed M. Saleh, Hamada AbdElgawad

**Affiliations:** 1 Department of Clinical Laboratory Sciences, College of Applied Medical Sciences, Al-Qurayyat, 2014, Jouf University, Saudi Arabia; 2 Department of Botany and Microbiology, Faculty of Science, Beni-Suef University, Beni-Suef 62521, Egypt; 3 Department of Botany and Microbiology, Faculty of Science, Cairo University, Giza 12613, Egypt; 4 Department of Biology, College of Science and Arts at Khulis, University of Jeddah, Jeddah 21959, Saudi Arabia; 5 Department of Biology, College of Science, University of Jeddah, Jeddah 21959, Saudi Arabia; 6 Department of Clinical Laboratory Sciences, College of Applied Medical Sciences, Jouf University, Sakaka, P.O. 2014, Saudi Arabia; 7 Botany Department, Faculty of Science, Suez Canal University, Ismailia 41522, Egypt; 8 Biology Department, Faculty of Science at Yanbu, Taibah University, King Khalid Rd., Al Amoedi, 46423, Yanbu El- Bahr, Saudi Arabia

**Keywords:** Actinobacteria, cell-free extract, drought stress, *Streptomyces*, osmoregulation

## Abstract

Drought stress is threatening the growth and productivity of many economical crops. Therefore, it is necessary to establish innovative and efficient approaches for improving crop growth and productivity. Here we investigated the potentials of the cell-free extract of Actinobacteria (Ac) isolated from a semi-arid habitat (Al-Jouf region, Saudi Arabia) to recover the reduction in maize growth and improve the physiological stress tolerance induced by drought. Three Ac isolates were screened for production of secondary metabolites, antioxidant and antimicrobial activities. The isolate Ac3 revealed the highest levels of flavonoids, antioxidant and antimicrobial activities in addition to having abilities to produce siderophores and phytohormones. Based on seed germination experiment, the selected bioactive fraction of Ac3 cell-free extract (F2.7, containing mainly isoquercetin), increased the growth and photosynthesis rate under drought stress. Moreover, F2.7 application significantly alleviated drought stress-induced increases in H_2_O_2_, lipid peroxidation (MDA) and protein oxidation (protein carbonyls). It also increased total antioxidant power and molecular antioxidant levels (total ascorbate, glutathione and tocopherols). F2.7 improved the primary metabolism of stressed maize plants; for example, it increased in several individuals of soluble carbohydrates, organic acids, amino acids, and fatty acids. Interestingly, to reduce stress impact, F2.7 accumulated some compatible solutes including total soluble sugars, sucrose and proline. Hence, this comprehensive assessment recommends the potentials of actinobacterial cell-free extract as an alternative ecofriendly approach to improve crop growth and quality under water deficit conditions.

## Introduction

Drought stress is one of the foremost environmental factors that negatively affect the quality and productivity of crops cultivated in (semi)-arid regions [1]. Recently, it was estimated that drought stress may cause reduction of food production in the range of 15-48% [2]. In this regard, it is expected that the next few years will see a double increase in dry land and 30% reduction in water availability [3]. This makes drought a major problem for crop productivity and hence, threatens food security for the rapidly growing population of the world [4]. Indeed, only twelve crops and fourteen animal species currently satisfy 90% of the food needs for the Earth’s inhabitants [5]. Among these crops, wheat, rice and maize are the major sources of food and energy for more than 50% of the global population [6].

Maize (*Zea mays* L.) is one of the most important cereals all over the world not only for nutrition but also for its economic and industrial applications. However, maize productivity is severely affected by drought conditions [7, 8]. Accordingly, it was reported that water shortages have induced sever reductions in maize productivity by 40% between the period from 1980 to 2015 [9]. The reduction in maize growth and productivity has been examined on physiological, biochemical and molecular levels [10]. The harmful, intracellular effects of drought stress on maize metabolism include excessive generation of free reactive oxygen species (ROS), oxidative damage to lipids and proteins, membrane permeability dysfunction, stomatal closure, enzyme inactivation and decline in assimilation of carbon [11, 12]. Therefore, it is essential to establish ecofriendly, cheap and potential solutions to sustain maize crop productivity under water deficit conditions [4, 13].

Currently, several approaches are being applied such as controlled watering, traditional breeding and using genetically modified drought-tolerant cultivars to improve maize growth under water shortage conditions. However, although promising results were reported under greenhouse condition, there have been some difficulties encountered in practice because they are technical and have high labor cost [10, 14]. Recently, treating plants with symbiotic microorganisms to improve the tolerance against drought stress in (semi)-arid regions has gained the attention of many researchers [15-17]. In this regard, there has been a huge increase of about 10% annually in the global market for microbial symbionts that enhance plant growth and increase yield under many stress conditions [1, 18]. Among these beneficial microorganisms, plant growth-promoting bacteria (PGPB) are considered one of the most promising solutions to improve crop growth and productivity under abiotic and/or biotic conditions [19]. Moreover, the PGPB, especially those isolated from dry regions, have been investigated to alleviate the negative effects of drought stress in many plants [20]. Actinobacteria are highly abundant microbial species of the soil microbiome and constitute about 10-50% of all species in different soil conditions [21]. Interestingly, the application of PGP Actinobacteria or their cell-free extracts provides considerable improvement in the growth and tolerance of crops like maize, soybean, rice and wheat under drought stress [16, 20].

The positive effect of Actinobacteria may also contribute to efficient use of water and minerals [22, 23], synthesis of essential plant hormones such as gibberellins (GA), auxin (IAA), cytokinins (CK) and salicylic acid (SA) [24], fixation of atmospheric nitrogen [25] and solubilization of phosphates [26]. They also increase generation of ROS scavengers and osmoprotectants [27] and enhance the activities of several enzymes involved in the plant antioxidant defense system [28].

The protective effects of Actinobacteria in maize under stressful environments were tested on a limited number of physiological and biochemical features [16, 29]. However, few works have addressed the simultaneous changes in growth, physiology and global metabolism of maize plant treated with Actinobacteria cell-free extract under drought conditions.

Thus, this work aims to address the comprehensive effects of cell-free extracts of several Actinobacteria isolates on the physiological responses and metabolic interface of maize experiencing drought. To our knowledge, this is the first study examining the global impact of Actinobacteria extract on maize under drought conditions.

## Materials and Methods

### Isolation of Actinobacteria

In order to isolate Actinobacteria, different soil samples were collected at a depth of 20 cm from a cultivated field in the Al-Jouf region (17°24'34.3 42°51'10.0), Saudi Arabia. Actinobacterial isolation was performed by soil dilution method reported earlier by Waksman [30] using glycerol-yeast agar medium (GYA: glycerol 5 ml/l, yeast extract 2 g/l, dipotassium phosphate 1 g/l, and agar 15 g/l) supplemented with nystatin (50 μg/l) as isolation medium. In brief, the soil suspension was prepared by mixing 1 g of dry soil with 9 ml sterile saline solution, which was then shaken well and heated at 50°C for 30 min. Then a ten-fold serial dilution of soil suspension up to 10^-8^ dilution was prepared. The last three dilutions of the soil suspension were inoculated into sterilized plastic plates followed by adding 20 ml of GYA medium. Then the poured medium was swirled slowly and incubated at 28°C for 7 days. Actinobacterial colonies with well-defined morphology were purified by sub-culturing the colonies on the same medium and then incubating for 7 days at 28°C. The pure isolates were preserved in slants containing starch casein agar medium at 4°C and in sterilized glycerol (20%) as suspensions at −20°C [31].

### Morphological, Physiological, Biological, and Biological Screenings of Actinobacteria

The identification of the obtained actinobacterial isolates was performed based on their morphological, physiological and biochemical features. The morphological investigation was performed using the light microscope according to the method of Shirling and Gottlieb [32]. Physiological and biochemical identification were done based on utilization of different nitrogen and carbon sources as well as activities of some enzymes following the method adopted by Williams *et al*. [33]. The antimicrobial activities were tested against the phytopathogenic bacteria *Pseudomonas syringae* and *Xanthomonas oryzae* following the standard disc diffusion methods as instructed by the Clinical and Laboratory Standards Institute (CLSI) for bacterial protocol MO2-A12 CLSI [34]. The average size of inhibition zones was used to determine the level of antibacterial activity. Streptomycin was used as a positive control with a concentration of 50 μg. The most potent bioactive actinobacterial isolates were further characterized and identified using Bergey’s Manual of Systematic Bacteriology Keys [33]. To test the plant growth potential of the potent actinobacterial isolate, the production of IAA and siderophore was carried out according to Gordon and Weber [35] and Schwyn and Neilands [36].

### Molecular Identification of the Most Potent Actinobacterial Isolates Using 16S rRNA Sequence Analysis

The selected actinobacterial isolate was then confirmed using molecular biology techniques including amplification of 16S rRNA and purification and sequencing of the amplicon as described before [37]. The obtained forward and reverse DNA sequence reads were used to produce contig sequence using DNAStar Lasergene software (version 7). The sequence of Actinobacteria was compared to references of 16S rRNA gene sequences of other actinobacterial isolates by the NCBI BLAST server.

### Preparation of Cell-Free Extract Fractions

Cells of the selected actinobacterium were harvested from a 3-day culture in GY liquid media by centrifugation at 6,000 ×*g*. The cells were sonicated in 80% ethanol (v/v), at 5% of the original culture volume, for 5 min at 130 W using an ultrasonic probe (Sonics, VCX130). The homogenate was centrifuged for 20 min at 12,000 ×*g* and the obtained supernatant was collected and used as a cell-free extract. The obtained cell-free extract filtrate was concentrated under reduced pressure in the rotatory evaporator till complete dryness to yield 0.1 g of crude material. The crude material was retained for further purification steps and sequentially divided into four fractions; F1 (n-hexane), F2 (acetone), F3 (n-butanol) and F4 (water). Employing thin-layer chromatography (TLC), the bioactive fraction (F2) was further fractionated on silica gel column, using hexane: acetone (100:0) to (5:1) as mobile phase that yielded 12 sub-fractions (F2.1- F2.12).

### Screening for the Bioactive Fractions

A homogenous lot of healthy maize seeds were surface sterilized using sodium hypochlorite (2.0%) for 20 min and then washed five times with distilled water. In order to screen for the bioactive fraction in the actinobacterial cell-free extract, groups of surface sterilized maize seeds, each of 100 seeds, were soaked in 1 ppm of cell-free extract or fractions (F1-4 and F2.1-2.12) for 4 h at 22oC, and then left to dry in an air cabinet. Another group of seeds were kept without treatment. After that, 20 uniform grains from each group were placed in each of five clean, sterilized Petri dishes (15 cm in diameter) lined with a filter paper. The effect of osmotic stress was induced by subjecting the non-treated and treated maize seeds to osmotic potential using 15% of polyethylene glycol (PEG 6000). The Petri dishes were incubated for four days in darkness at 25°C. After seven days, the seedling fresh mass was determined, and the percentages of germination were estimated using the following equation: Germination percentage = Number of normal seedlings/Number of total planting seeds × 100.

### Isolation and Identification of the Bioactive Compounds in the Most Active Fraction (F2.7)

TLC analysis revealed that sub-fraction (F2.7) mainly contained one active compound. This fraction was further purified using high-performance liquid chromatography (HPLC), (Shimadzu class-LC 10 AD chromatograph coupled with Shimadzu SPD-10 AUV-VIS, phenomenex C18, 25 cm 4.6 mm, 5 Mm particle size). The purity of this sub-fraction was verified by UV spectrum analysis, which showed one main peak with maximum absorption at 250 nm. The purified compound was further identified by ^1^H and ^13^C nuclear magnetic resonance analysis (NMR).

### Greenhouse Experiment

A group of sterilized seeds were soaked in the prepared cell-free extract or the best bioactive fraction (F2) prior to cultivation in the soil. Another group of seeds were soaked in culturing medium without Actinobacteria as a control. Plants, treated or non-treated, were subjected to severe drought stress. The plants in the control groups were re-watered daily up to 60% (soil water content (SWC)). In drought treatments, the water contents were adjusted after sowing to 30% SWC (severe stress, leaves wilting during the day). The growth conditions of the greenhouse were set at 21°C, with a 16 h photoperiod and at 60% humidity. At the end of the experiment, treated and non-treated plants were collected for morphological and biochemical analyses.

### Estimation of the Photosynthesis Rate

The rate of photosynthesis was determined to fully expanded leaves [7] using the portable photosynthesis system (LI-COR LI-6400, USA) and the photosynthesis rate was expressed by μmol/CO_2_/m^-2^/s^-1^. A minimum of 5 min of leaf equilibration was set at each step before data were logged. The system leaf chamber conditions were set at 400 or 620 ppm CO_2_, 22oC (block temperature) and saturated photon flux density was 1500 μmol/m^-2^/s^-1^.

### Stress Markers

Lipid peroxidation was determined by quantifying the fatty acid oxidation-produced malonaldehyde (MDA) according to the thiobarbituric acid assay [38]. Protein oxidation was assessed via carbonyl quantification [39]. Whereas, xylenol orange method was employed to measure hydrogen peroxide (H_2_O_2_) in tricarboxylic acid cycle (TCA; 0.1%) extract of plant samples [40].

### Metabolite Profiling

The contents of carbohydrates including some individual sugars, organic acids, amino acids and fatty acids were estimated using (HPLC) or gas chromatography mass spectrometry (GC/MS) analyses according to the methods described in our previous investigation [41]. The identification and quantification of individual compounds were achieved by comparing the peak area with that of calibration curve of the corresponding standards and referring to NIST(05) and Golm Metabolome (http://gmd.mpimp-golm.mpg.de) databases. Extraction and quantification of the total concentration of sugars was carried out following Nelson’s colorimetric method as described in AbdElgawad *et al*. [42].

### Quantitative Estimation of Individual Sugars

The dried powder obtained from maize leaves was extracted in acetonitrile (50%, v/v). Sugars profile in the clear extract was quantified by HPLC analysis. Mobile phase (acetonitrile and water at 75:25 ratio (v/v)) was applied at a flow rate of 1 ml/min at 30°C [41]. To identify sugars, their retention times were compared with corresponding standards. The levels of each sugar were determined by using a calibration curve.

### Organic Acids Profile Analysis

Maize dried powdered tissues of known weight were homogenized in butylated hydroxyanisole (0.3%, w/v) and phosphoric acid (0.1%) and by using a MagNA Lyser instrument. Organic acids were estimated by HPLC (Supelcogel C-610H column), a UV detection system operated at 210 nm (LaChromL-7455 diode array, LaChrom, Japan). Next, 0.1% of phosphoric acid (v/v) was applied as mobile phase (0.45 ml/min flow rate) [41]. The levels of each organic acid were estimated by calibration curve for each corresponding standard.

### Amino Acids Profile

The amino acids in maize dried powdered tissues were extracted in 80% aqueous ethanol using a MagNA Lyser (Roche, Belgium) [43]. The clear supernatant was obtained by centrifugation for 20 min at 14,000 ×*g*, then evaporated to dryness by lyophilization under vacuum, and the precipitate was re-suspended in 5 mL chloroform. High purity deionized water was used to re-extract the residue then was clarified by centrifugation. In order to obtain the aqueous phase containing the amino acids, supernatants were re-mixed with chloroform then centrifuged, decanted and filtered through Millipore membrane filters (0.2 μM diameter). The amino acids were separated and quantified by Waters Acquity UPLC-tqd System (Milford, USA) coupled with the column BEH amide (2.1 × 50).

### Isolation and Analysis of Fatty Acids

For the fatty acids’ isolation, identification and quantification, the method of Torras-Claveria *et al*. [44] was used. For the preparation of lipophilic fractions, dried powdered leaves (200 mg) were extracted in aqueous methanol (50%) at 25°C. Then GC/MS analysis was carried out on a Hewlett Packard 6890, MSD 5975 mass spectrometer (Hewlett Packard, USA) and the column HP-5 MS (30 m × 0.25 mm × 0.25 mm) was connected, and the internal standard used was nonadecanoic acid. For identification of the fatty acids, the NIST(05) and Golm Metabolome (http://gmd.mpimp-golm.mpg.de) databases were used.

### Quantitative Analysis of Antioxidants

The antioxidant glutathione and ascorbate content was extracted then determined with reversed-phase HPLC according to Potters *et al*. [45]. The Polaris C18-A column (100 × 4.6 mm, 3 μm particle size; 40°C) was utilized for separation. The detection was performed using an in-line diode array detector (DAD). In order to determine total glutathione and total ascorbate, the maize extract was reduced with M DTT (0.04) for 12 min at room temperature before application to column. Tocopherols were obtained by hexane extraction according to Siebert [46]. Tocopherols were separated and quantified by HPLC (normal phase) conditions (Particil Pac 5 μm, 250 mm, i.d. 4.6 mm), and the internal standard was 5,7-Dimethyltocol (DMT; 5 ppm). The carotenoids were separated using a reversed-phase HPLC on a C18 column. The mobile phase used consisted of solvent A (acetonitrile:methanol:water; 81:9:10) and solvent B (methanol:ethyl acetate; 68:32) and flow rate was 1.2 ml/min at ca. 70 bar. The detection at 420, 440, 462, 660 nm was done by DAD detector (Shimadzu SPD-M10Avp). The calibration curve was used to determine the concentration. For quantification of phylloquinone, HPLC ((reversed-phase coupled with RP18 column (Eurosphos-100, 250 × 4.6 mm)) was used according to the method described by Jakob and Elmadfa (1996). The elution system (methanol:dichloromethane; 90:10) was supplemented with methanolic solution containing ZnCl2, sodium acetate and acetic acid (1.37, 0.41, 0.30 g, respectively) per liter of the mobile phase. Phylloquinone was measured by fluorescence detector (excitation, 243 nm; emission, 430 nm) and the internal standard used was menaquinone-4.

### Total Antioxidant Activity

The maize tissue extracts’ scavenging activity of free radicals was assayed by two methods, 2,2-diphenyl-1- picryl-hydrazyl‐hydrate (DPPH) and fluorescence recovery after photobleaching (FRAP) [47]. The extracts were prepared by grinding fresh maize tissues (300 mg) in 3 ml 80% (v/v) ice-cold ethanol. The DPPH assay was performed by mixing each extract with 0.5 ml of DPPH solution (0.25 mM in 95% ethanol). The mixture was shaken well then allowed to stand for 30 min at room temperature. Thereafter, 2 ml of double distilled water was added, and the absorbance was measured at 517 nm to calculate the percentage of inhibition. For the FRAP assay, the freshly prepared reagent was dispensed in the micro-titration plate and mixed extracts. The incubation was for 30 min at 37°C, the absorbance was measured at 593 nm (micro-plate reader, Synergy Mx, Biotek Instruments Inc., USA). The antioxidant capacity was calculated via Trolox standard-curve quantification using concentration up to 650 μM.

### Statistical Analyses

Statistical analyses of data were carried out via analysis of variance (one-way ANOVA) from the SPSS statistical program (SPSS Inc., USA). Tukey’s test (*p* ≤ 0.05) was used as the post-hoc test for separations of mean. Different letters beside mean values indicate significant difference, while values sharing at least one common letter are not significantly different. Each experiment was done in three replicates.

## Results and Discussion

### Morphological and Biochemical Identification of the Isolated Actinobacteria

Three actinobacterial strains (Ac3, Ac6 and Ac11) were isolated and purified from the rhizosphere of cereal plants cultivated fields in Jouf city (Saudi Arabia). The isolates were classified based on morphological and biochemical features. Microscopic examination revealed that the three isolates belong to the genus *Streptomyces.* At the morphological level, isolate Ac11 was characterized with aerial mycelium, branched hyphae, spiral spore chains and/or long rectiflexibiles. Pigmentation was observed in isolates Ac3 and Ac11 but no diffusible pigment was produced for isolate Ac6 on starch nitrate medium. The screened *Streptomyces* species revealed different substrate colors, the isolate Ac3 substrate mycelium was gray, isolate Ac11 was yellow while no color was observed for isolate Ac6 (Table 1). The isolated species exhibited different capabilities to grow on different types of carbon and nitrogen media and produced different enzymes.

This result is consistent with the variations in the biochemical and morphological features reported among different Actinobacteria strains isolated from rhizosphere of palm tree [47] and with the previously characterized five Actinobacteria species isolated from the rhizospheric soil of cereals [20]. It is well known that *Streptomyces* is the largest genus of Actinobacteria and family *Streptomycetaceae*, which usually inhabit soil and are represented by more than 500 species [48, 49].

### Antioxidant and Antibacterial Activities of Actinobacterial Cell-Free Extracts

**Fig. 1:**
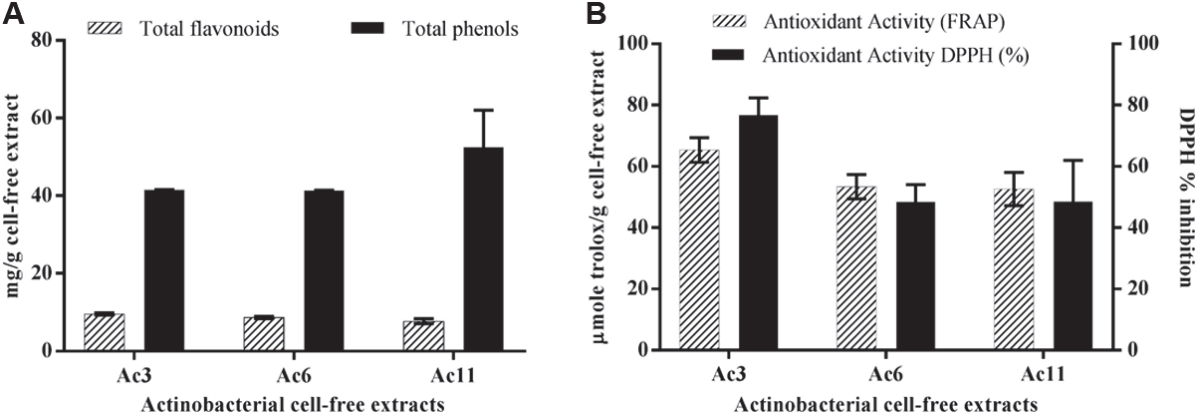
Assessment of the crude actionobacterial cell-free extracts. (**A**) Flavonoid and phenolic contents (mg/g cell- free extract), (**B**) antioxidant capacity as analyzed by FRAP, μmole Trolox/g extract as well as DPPH percentage of inhibition.

**Fig. 2:**
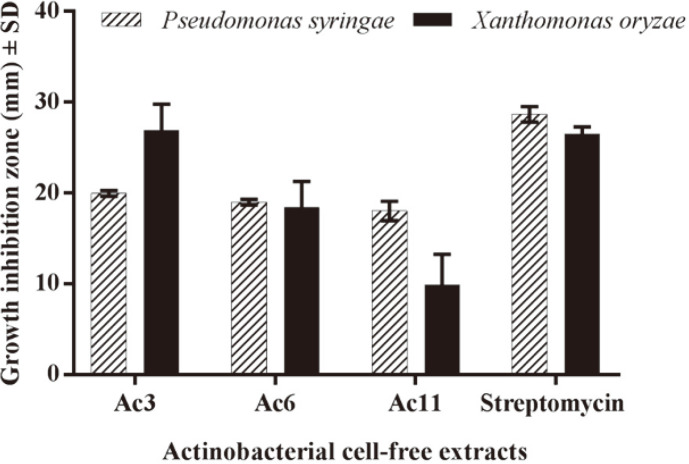
The antibacterial activities of crude actinobacterial cell-free extracts against the phytopathogens *Pseudomonas syringae* and *Xanthomonas oryzae*. Streptomycin was used as a positive control with concentration 50 μg.

In order to choose the most active actinobacterial cell-free extracts, antibacterial and antioxidant activity were first tested. Results showed considerable antioxidant activities using both DPPH and FRAP assays in all tested isolates, which closely matched with their higher flavonoid contents (Fig. 1). Among tested isolates, Ac3 revealed the greatest antioxidant capacities as well as antibacterial activities comparable to positive control, giving zone of inhibition diameters of 19.974 ± 0.325 and 26.947 ± 2.839 mm against *Pseudomonas syringae* and *Xanthomonas oryzae*, respectively (Fig. 2).

Additionally, there were reported potential antioxidant activities in bioactive compounds extracted from *Streptomyces lydicus* A2, as well as antibacterial activities against *Staphylococcus aureus* and *Bacillus cereus* [50]*.* Furthermore, the purified fraction *of Nocardiopsis alba* isolated from mangrove soil has shown DPPH radical scavenging effect greater than 50% at concentration of 50 µg/ml [51]. Moreover, a positive correlation between the phenolic compounds and the higher antioxidant and the biological activities was reported by Dholakiya *et al*. [52] for marine actinobacterium (*Streptomyces variabilis* RD-5). Taken together, these results could reflect the capabilities of the tested actinobacterial extract to alleviate the abiotic and/or biotic stress in plant.

### Molecular Identification and Phylogenetic Analysis of the Most Active Actinobacteria

**Fig. 3:**
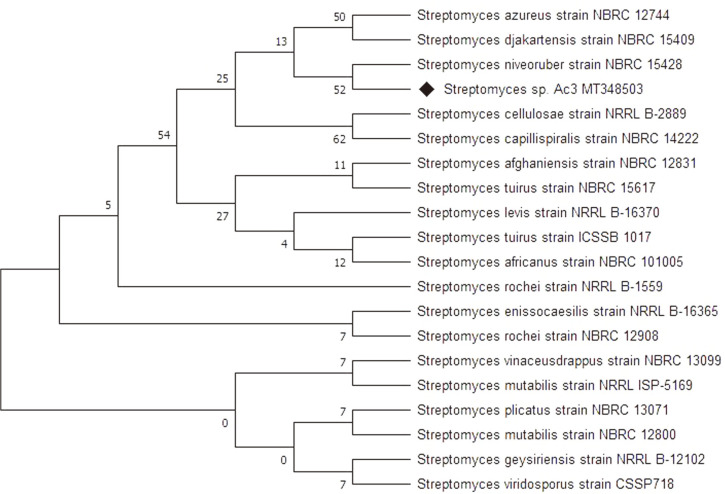
The phylogenetic tree constructed by the maximum likelihood method using MEGAX software for the 16S rRNA sequences of *Streptomyces* sp. AC3. Numbers at nodes represent the percentage values given by 1,000 bootstrap analysis samples.

The identity of the most active actinobacterial isolate Ac3 was confirmed by sequencing the 16S rRNA gene using the universal primers 27 and 1492R. The amplified sequence exhibited a high degree of homology, showing 99.79% similarities with 16S rRNA gene from many Streptomyces species such as *Streptomyces rochei*, *Streptomyces mutabilis*, *Streptomyces geysiriensis* and *Streptomyces enissocaesilis*. The obtained sequence was registered into the GenBank database under the accession number MT348503. The MEGAX software was used to build the phylogenetic relationship with the related *Streptomyces* strains by maximum likelihood method and evaluated by bootstrap analyses based on 1,000 samples (Fig. 3).

### Characterization of the Most Active Actinobacteria Cell-Free Extract

Among the tested isolates, the Ac3 cell-free extract was further characterized. The profile of flavonoids and the contents of the phytohormones and siderophores were measured in the candidate isolate Ac3 (Table 2). The results showed that isoquercitrin was the predominant flavonoid with a concentration of 8.361 ± 0.67 mg/g cell-free extract followed by apigenin (7.489 ± 0.6 mg/g cell-free extract). Moreover, a remarkable production of hormones (IAA, ABA and GA) and of siderophores (*e.g*., catechol and salicylate-types) was recorded.

Similarly, Banerjee *et al*. [53] demonstrated a remarkable increase in rice plant root and shoot length after the inoculation with bacterial or fungal isolates that were able to produce IAA, siderophore and ammonia, and the ability to solubilize divalent phosphate. Studies by other researchers have declared that rhizobacteria induced- plant growth has pronounced, positive effects on root and shoot weight, plant growth and yield [54, 55].

### F2.7 is the Most Effective Sub-Fraction in Enhancing Maize Tolerance to Drought

**Fig. 4:**
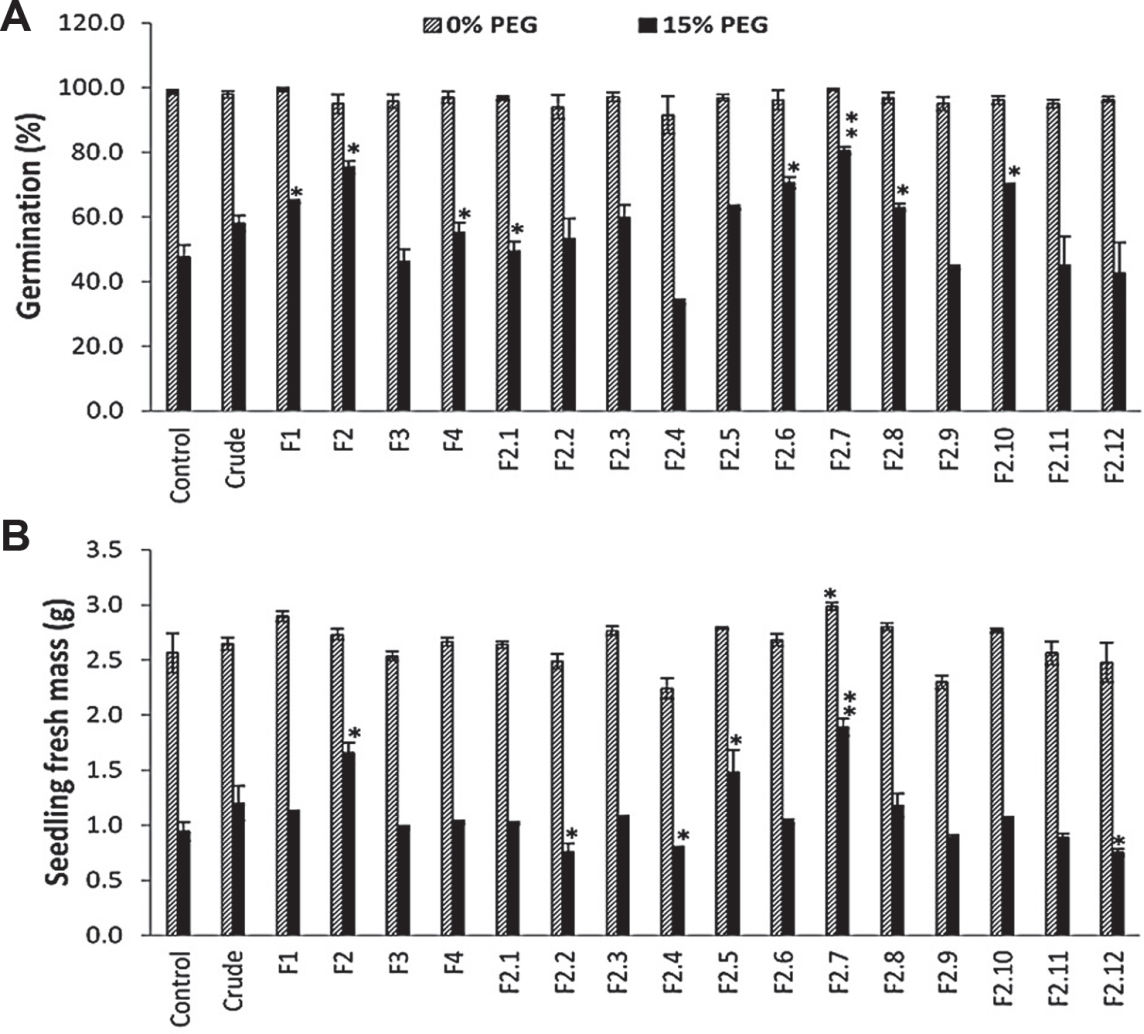
Impact of crude actinobacterial cell-free extract or its fractions (F1-4 and F2.1-2.12) on germination percentage. (**A**) Seedling fresh mass (**B**) of maize grown under normal (0% polyethylene glycol, PEG) or osmotic stress conditions (15% PEG). Asterisks indicate significant changes (*p < 0.05; **p < 0.01) compared to control.

In order to select the most active fraction of the cell-free extract of Ac3, a seed germination experiment was conducted using 4 fractions (F1-F4) under induced osmotic pressure (15% PEG). The results indicated that F2 increased the percentage of germination compared to control without treatment under the same conditions (Fig. 4). In addition, the highest maize seedling FW was recorded for the same fraction F2. Further fractionation was conducted for the best active fraction F2 and 12 sub-fractions were obtained. All sub-fractions were evaluated, and the results revealed that sub-fraction F2.7 was the best in promoting the germination percentage and seedling fresh mass of maize grown under both normal and osmotic stress conditions (Fig. 4). Therefore, F2.7 was further evaluated. The sub-fraction (F2.7) mainly contained one active compound which was identified by ^1^H and ^13^C NMR. The ^1^H NMR spectra signals were: 1H-NMR (dissolved in DMSO-d6) 6.20 (1H, d, J = 2.1 Hz, H-6), 6.40 (1H, d, J = 2.1 Hz, H-8), 6.86 (1H, d, J = 8.4 Hz, H-5'), 7.57 (1H, dd, J = 8.5 and 2.3 Hz, H-6'), 7.71 (1H, d, J = 2.4 Hz, H-2'), 5.41 (d, J= 7.6 Hz, H-1-glc). Also, the signals of 13C-NMR (dissolved in DMSO-d6), 157.32 (C-2), 134.21 (C- 3), 178.8 (C-4), 162.441 (C-5), 99.85 (C-6), 165.61 (C-7), 94.58 (C-8), 157.61 (C-9), 104.13 (C-10), 122.17 (C-1'), 116.04 (C-2'), 145.64 (C-3'), 149.19 (C-4'), 118.02 (C-5'), 122.95 (C-6'), 103.31 (C-1''), 60.7 (C-6''), 69.12 (C-4''), 75.3 (C-2''), 77.01 (C-3''), 78.07 (C-5''). Accordingly, the purified compound was identified as isoquercetin.

### Effect of F2.7 on Maize Growth and Photosynthesis Under Drought Conditions

Desiccation is known to threaten plant life through impairing essential processes like photosynthesis and hence, induces plant growth decline [27]. This fact is very consistent with the current results, where drought caused marked decrease in maize fresh weight (FW) and dry weight (DW) by 53.5% and 48.1%, respectively, as compared to well-watered plants (Table 3). Interestingly, actinobacterial Ac3 cell-free extract F2.7 fraction induced significant increase in both FW and DW by 96.6% and 52.4%, respectively, compared to non-treated maize plants under drought. Moreover, the net photosynthesis reduced markedly by 44.2% under drought condition. On the other hand, this reduction was significantly recovered by 52.2% due to F2.7 treatment, relative to the non-treated plants (Table 3).

The same observed reduction in biomass and photosynthesis was investigated by Khan *et al*. [58, 59] in maize grown under drought conditions. They attributed the reduction in growth and photosynthesis under drought to the ROS-induced oxidative burst. Additionally, it was stated that drought impairs photosynthesis by membrane disorganization, stomatal closure and inactivation of enzymes [60, 61]. On the other hand, the potential of actinobacteria to alleviate the inhibitory effects of drought on the growth (biomass accumulation) and physiology (photosynthesis) of maize was tested in many crops. In this regard, Hozzein *et al*. [62] found marked elevation in growth and photosynthesis of barley, wheat, maize, sorghum and oat plants treated with Actinobacteria. Also, Dicko *et al.* [63] and Govindappa *et al*. [64] reported significant increase in biomass of maize inoculated with PGPB as compared to non-inoculated plants. Such increase in growth and photosynthesis due to actinobacterial treatment has been attributed to enhancement in photosynthetic pigment synthesis and nitrogen accumulation [47, 62] and activation of enzymes necessary for chlorophyll biosynthesis and other metabolic pathways [65, 66].

### The Effects of F2.7 and Drought on Maize Primary Metabolism

It is well known that photosynthesis is the most vital process in plant metabolism, whereas carbohydrates are the main energy source essential for all other metabolic pathways. Thus, due to its positive effect on photosynthesis, we assumed that treating maize with Actinobacteria cell-free extract may alter its metabolism and consequently impact its behavior under water deficit conditions. Therefore, the accumulation of many primary and some secondary related metabolites was estimated in treated and non-treated maize grown in drought and normal irrigation conditions. The levels of carbohydrates, amino acids and organic acids were assessed in both treated and non-treated maize under drought and normal conditions (Table 3). For carbohydrates, the accumulation was increased under drought conditions, especially the levels of total soluble sugars, total carbohydrates and starch, which were increased significantly by 60%, 30.2% and 28.5%, respectively, as compared to control plants. The F2.7 fraction of actinomycetes caused an increase in all fractions of sugar, and sucrose was significantly increased by 70.2% compared to the non-treated plants under water deficit.

Such impact of Actinobacteria on carbohydrate accumulation was correlated with the observed enhancement in photosynthesis (Table 3). The improved photosynthesis combined with increase in the accumulation of carbohydrate was reported before [7]. In addition, accumulation of sugars like glucose, fructose and sucrose might activate the TCA cycle and hence, increase cycle intermediates like some organic acids [67, 68]. In line with this, comparison between the organic acid in treated and non-treated maize plants under drought conditions indicated significantly higher concentrations of oxalic, isobutyric, citric, and fumaric by 25.4%, 67.16%, 43.1% and 43.1%, respectively (Table 3). This accumulation of carbohydrates and organic acids was observed in chickpea inoculated with PGPR by Khan *et al.* [58, 59] and maize treated with Actinobacteria after the plants were subjected to drought (Saleh *et al*., 2019). Impaired photosynthesis, changes in carbohydrates and organic acids biosynthesis are typical activities of drought-stressed plants [69].

Under abiotic stress environment, the plant amino acids are accumulated to cope with the stress because amino acids are the precursors of protein and some amino acids act as osmoprotectants or antioxidants [70]. Overall, the results obtained revealed that the levels of glutamine and asparagine were increased in non-treated maize under drought condition by 16.8% and 64.2%, respectively. Whereas, the rest of the amino acids were accumulated significantly in the treated plants under same conditions (Table 4). Induced amino acid levels in treated maize may be attributed to the protective role of actinobacterial cell-free extract in improving plant photosynthesis and growth. In this context, drought-increased amino acid levels have been previously reported [71]. Additionally, the fatty acid profile in maize was analyzed in treated and non-treated plants under normal and drought treatments. It is well known that fatty acids are important not only as membrane structural components or storage compounds but also, for synthesis of plant hormones such as jasmonates that enhance plant tolerance against drought stress [72, 73]. In this regard, data revealed that drought stress caused significant elevation of the majority of the fatty acids except for Eicosadienoic (C20:2) and Hexadecanoic (C16:0) that were reduced significantly by 8.6% and 35%, respectively, as compared with well-watered maize plants. The cell-free extract fraction induced marked rise in fatty acids content in treated maize plants under both normal and water deficit irrigation (Table 3). Similar data were obtained in maize treated with Actinobacteria under drought stress [20]. This suggests that the secondary metabolites produced by the actinomycetes played the dominant role.

### Actinobacterial Fraction F2.7 Treatment Enhanced Maize Tolerance Against Drought Induced Osmotic Stress

Osmotic adjustment including osmolyte accumulation is one of the chief strategies through which plants maintain proper water uptake and cell turgidity to tolerate water deficit conditions [74, 75]. In order to cope with osmotic stress, the plant cells accumulate compatible osmolytes such as low molecular mass biomolecules including glycine, proline, betaine and soluble and alcoholic sugars [76] to protect the cell organelles from oxidative damage triggered by drought [77].

In line with this, the cell-free extract fraction F2.7 induced the significant increase in the total soluble sugars, sucrose, proline, arginine and glycine betaine under water deficit conditions, compared to non-treated plants under the same conditions. Similarly, Tomczyk and Gudej [78] observed a marked increase in the content of soluble sugars in cucumber plants treated with rhizobacteria under drought.

### Treatment with F2.7 Maintained the Redox Homeostasis and Mitigated Drought-Induced Cell Damage

Accumulation of ROS and its related oxidative damage is a characteristic of plants cultivated under drought stress [27]. The lack of efficient antioxidant defense systems to maintain redox balance inside the cell is correlated to damage induced in the essential cell compounds, especially membrane lipids and proteins and hence, cell injury [79]. The obtained data showed a significant accumulation of H_2_O_2_, protein carbonyls and lipid peroxides (MDA) by 38.6%, 145.7% and 139%, respectively, in non-treated maize in response to drought condition (Table 4).

Plants scavenge ROS and hence, tolerate oxidative stress, through production of low-molecular-weight water soluble antioxidants such as ascorbate (ASC) and glutathione (GSH) and the lipid soluble tocopherol, polyphenols and carotenoid compounds [80, 82]. In this regard, the recorded data (Table 4) revealed that drought stressed maize plants treated with cell-free extract fraction F2.7 have an enhanced redox homeostasis designated by low concentrations of stress markers (H_2_O_2_, protein carbonyls and MDA) and higher TAC (FRAP), as compared with the non-treated ones. These improved antioxidants in treated maize plants could be attributed to the improvement in the accumulation of biomolecular antioxidants. Consistent with this hypothesis, the current results showed marked accumulation in ASC, GSH and tocopherols level in treated maize under drought, relative to the non-treated plants. Similarly, Selim *et al*. [20] and Vardharajula *et al*. [82] observed accumulations in the levels of molecular antioxidants and improvement of plant redox homeostasis after treatment with Actinobacteria under drought stress.

In conclusion, using secondary metabolites of PGPB is an ecofriendly and economically effective approach to improve the growth of maize plants under normal and drought conditions. The potential of Actinobacteria extract in improving maize growth and tolerance against drought may be ascribed to phytohormones and siderophores which enhance root health and therefore, facilitate the absorption of minerals and nutrients from soil. Furthermore, the positive indirect impacts of Actinobacteria antioxidant and osmotic-related metabolites may be due to the induction of plant ROS scavengers and osmoprotectants such as sugars, amino acids, phenolics and flavonoids. Besides, the effective actinomycetes fraction F2.7 not only enhances maize drought tolerance but also improves the nutritional quality of the crop through increasing the contents of some vital compounds like fatty acids and carbohydrates. Thus, the obtained results recommend utilization of cell-free extract of Actinobacteria as an effective alternative tool for enhancing both growth and performance of important crops grown under abiotic conditions.

## Figures and Tables

**Table 1:** Morphological characterization , nitrogen and carbon source utilization, and biochemical activities of selected actinomycete isolates (Ac3, Ac6 and Ac11).

	Isolates	Ac3	Ac6	Ac11
*Spore chain*	Aerial mycelium	+	+	+
	Pigmentation	+	-	+
	Spiral	+	+	+
	Rectiflexibiles	-	-	+
	Verticillat	-	-	-
*Substrate*	Yellow	-	-	+
	Orange	-	-	-
	Gray	+	-	-
	Red	-	+	-
*N source utilization*	L-Cysteine	+	+	+
	L-Phenylalanine	-	+	+
	L-Histidine	+	-	+
	L-Lysine	+	+	+
	L-Asparagine	+	+	-
	L-Arginine	-	+	+
	L-proline	+	+	-
	L-Valine	+	+	+
	Tyrosine	+	+	+
*C source utilization*	D-fructose	+	+	-
	D-glucose	+	-	-
	Sucrose	-	+	+
	Maltose	+	-	-
	Raffinose	-	+	+
	Lactose	+	+	-
	Galactose	-	+	+
	Meso-Inositol	+	-	-
	Celullose	+	-	+
	Xylose	-	+	+
	Dextran	+	+	-
*Enzymes activity*	Catalase	+	+	+
	Peroxidase	+	+	+
	Starch hydrolysis	-	+	-
	Gelatin liquefication	+	+	+
	Casein hydrolysis	-	-	+
	Lipolysis	+	+	+
	Citrate utilization	+	+	+
	Nitrate reduction	+	-	-
	Urease	+	+	+
	H_2_S Production	+	+	+
	DNase	+	+	+

The + and – signs indicated the presence and absence, respectively.

**Table 2:** The contents of flavonoids, hormones and siderophores produced by the selected actinomycete (Ac3).

Parameter	Ac3
Quercetin	1.143±0.09
Quercetrin	1.915±0.15
Luteolin	0.901±0.12
Apigenin	7.489±0.6
Isoquercetrin	8.361±0.67
Rutin	0.847±0.07
Ellagic acid	0.417±0.01
Velutin	0.357±0.03
Naringenin	1.232±0.1
Genistein	1.885±0.15
Daidzein	2.082±0.17
Fisetin	3.847±0.31
O-hydroxydaidzein	1.033±0.08
IAA-Me	1.1598
13C6-IAA-Me	
ABA	0.1098
d6-ABA	
GA	0.1663
Sidephore Catechol	9.1961
Sidephore Salicylate	7.653

Values are the average of 3 replicates (mean ± SD).

**Table 3:** Effect of maize treatment with actinomycete (Ac3) bioactive sub-fraction F2.7 on the biomass, photosynthesis, the contents of carbohydrates, organic acids, amino acids and fatty acids under normal and drought condition compared to non-treated control plants.

Parameter	Control	Control+F2.7	Drought	Drought +F2.7
**Biomass and Photosynthesis**				
FW (g/plant)	5.08±0.09a	4.13±0.97ab	2.36±0.78c	4.64±0.74d
DW (g/plant)	0.81±0ab	0.91±0.13a	0.42±0.07d	0.64±0.09c
**Photosynthesis (umoles CO_2_ m^-2^ leaf area S^-1^)**	8.14±1.09a	7.21±1.08a	4.54±0.81c	6.91±0.96b
**Carbohydrates**				
Glucose	1.82±0.09c	1.66±0.07ab	2.88±0.6a	3±1.1a
Fructose	1.09±0.13a	1.14±0.17a	2.92±0.2a	3.4±0.2a
Sucrose	1.93±0.17a	2.07±0.98a	3.02±0.22a	5.2±0.2b
Soluble sugars	9.06±1.51b	9.1±0.7a	14.51±1.88a	19.8±1.1a
Starch	27.83±1.35c	30.19±0.23b	35.77±0.9ab	39.3±2a
Total Carbohydrates	62.07±3.05c	70.81±4.17ab	80.81±6.29a	94±6.4a
**Organic acids**				
Oxalic	3.92±0.93b	4.34±0.26ab	4.75±0.41b	7.94±1.08a
Malic	25.07±1.23b	19.6±0.61b	17.81±0.99ab	42.72±1.36a
Succinic	2.84±0.27b	2.47±0.66b	4.71±0.22ab	6.35±0.46a
Citric	5.21±0.16cd	8.19±0.68c	15.91±1.21b	19.95±0.73a
Isobutyric	5.37±1.42ab	5.33±1b	4.78±0.41b	6.84±0.83a
Fumaric	8.67±1.01c	9.25±0.31c	12.06±1.07b	17.04±1.03a
**Amino acids**				
GlutamIic acid	30.24±ab	34.48±ab	44.73±b	47.98±a
Glutamine	121.87±c	132.11±c	142.35±b	162.6±a
Lysine	7.56±b	6.13±ab	4±b	4.26±a
Alph-keto glutaric acid	0.05±b	0.06±b	0.09±ab	0.11±a
Histidine	1.15±a	0.95±ab	0.74±a	1.54±a
Alanine	34.75±ab	50.13±b	65.52±b	80.9±a
Arginie	1.4±ab	1.71±ab	2.03±ab	2.83±a
Ornithine	0.1±a	0.1±a	0.11±a	0.87±a
Proline	0.8±ab	0.77±ab	1.12±b	1.29±a
Asparagine	1.2±c	1.61±b	1.97±b	2.27±a
Isoleucine	0.23±a	0.32±a	0.31±a	0.47±b
Leucine	0.18±a	0.23±a	0.41±a	0.39±b
Methionine	0.59±a	0.5±ab	0.91±a	1.33±b
Threonine	0.226±a	0.106±a	0.67±a	0.3±b
Valine	1.5±a	1.93±a	1.81±a	2.92±b
Serine	0.28±a	0.35±a	0.43±a	0.5±b
Phenylalanine	0.73±a	0.71±b	0.5±ab	0.38±a
Glycine	1.01±a	0.7±a	0.4±a	0.1±b
Aspartaat	0.04±b	0.04±c	0.06±b	0.08±a
Cystine	0.19±a	0.23±ab	0.26±a	0.3±a
Tyrosine	1.01±ab	0.78±b	1.56±a	2.33±a
**Fatty acids**				
Dodecanoic (C12:0) )	1647.2±147.03b	2849.6±357.79a	3051.9±168.2a	4254.2±192.85c
Tetradecanoic (C14:0	716.7±47.93a	821.1±6.6a	925.4±91.63b	1029.8±81.55b
Pentadecanoic (C15:0)	136.8±5.73a	164.2±10.96a	191.6±7.22bc	219±8.72d
Hexadecadienoic (C16:2)	1303.6±185.01a	1540.8±103.96b	1778±176.79b	2215.3±119.77c
Heptadecanoic (C17:0)	2941.1±295.31b	4901.2±392.5a	8861.3±203.28b	11821.4±265.19b
Octadecanoic (C18:0)	110.6±12.7a	97.4±7.1a	124.2±8.09b	171±6.6c
Eicosanoic (C20:0)	2498.4±222.98b	2562±372.2a	3625.6±212.7b	4689.2±241.9c
Docosanoic (C22:0)	167.5±11.21a	175.3±13.37a	223±20.42b	310.7±3.36c
Tricosanoic (C23:0)	16.8±0.75a	16.3±1.12a	15.7±0.87a	15.2±0.87b
Pentacosanoic (C25:0)	530.8±60.88a	517.5±44.32a	774.2±38.1b	650.8±31c
Hexadecatrienoic (C16:3)	506.3±71.96a	619.9±99.6b	703.6±56.03b	847.2±57.52c
Octadecenoic (18:1)	174.4±11.7a	151.2±21.25a	228±19.67b	304.7±3.74c
Octadecatrienoic (C18:3)	984.2±63.25a	996±86.78a	1296.8±64.86b	897.5±85.53c
Eicosadienoic (C20:2)	362.2±29.13a	346.7±18.55a	331.1±23.28b	315.5±19.42b
Tetracosenoic (C24:1)	345.5±74.82a	491.2±49.3b	536.9±3.86b	427.4±28.39c
Hexadecanoic (C16:0)	8395.1±147.72a	9233.4±506.22ab	5471.7±124.93b	4010.1±408.57c
Hexadecanoic (C16:1	283.2±43.08a	231.8±61.38a	380.4±41.46ab	379±37.1c
Octadecadienoic (C18:2)	32±2.12a	32.6±0.25a	41.3±3.86a	33.9±0.62a
Tetracosanoic (C24:0)	210.8±8.96a	221.9±14.82a	333±9.59b	244.1±10.83c
Hexacosanoic (26:0)	42.3±4.86a	55.1±3.98a	67.9±3.24b	80.7±6.1a

Values are the mean of 3 replicates ± SD Different letters represent significant differences between the treatments in each group as analyzed by Duncan’s test *p* < 0.05.

**Table 4:** Effect of maize treatment with actinomycete (Ac3) bioactive sub-fraction F2.7 on contents of lipid peroxidation product (MDA), protein oxidation, total peroxides (H_2_O_2_), ascorbate (ASC), glutathione (GSH), tocopherols, and glycine betaine as well as antioxidant power (FRAP) in maize compared to nontreated control plants under normal and drought condition.

Parameters	Control	Control+F2.7	Drought	Drought+F2.7
MDA (mg/g)	8.1±0.08a	7.71±0.97a	19.35±1.03c	9.07±1.67b
H_2_O_2_ (umol/g)	450.61±0a	459.59±0.13a	624.45±0.07c	457.96±0.09b
Protein oxidation (mg carbonyl/gFW)	1.4±1.09a	1.69±0.88a	3.44±0.81b	2±1.32a
ASC	2.2±0.12a	3.21±0.1a	4.65±0.2b	6.3±0.3b
GSH	0.21±0.02a	0.3±0.1a	0.66±0.02b	0.6±0ab
Tocopherols	2.23±0.31a	2.58±0.34a	3.46±0.35b	4±0.4a
FRAP	16.76±1.42a	22.53±1.41a	30.88±1.15c	41.5±1b
Glycine betaine	4.16±0.26a	3.93±0.25ab	7.05±0.92d	5.9±0.6c

Values are the mean of 3 replicates ± SD. The different letters indicate significant differences between the treatments in each group as analyzed by Duncan’s test *p *< 0.05.
